# Inter-generational transmission of psychological capital for adolescents: the mediating role of community social capital and the moderating role of spatial stratification

**DOI:** 10.3389/fpubh.2026.1728505

**Published:** 2026-02-18

**Authors:** Qingyu Bu, Mei Peng, Zhuojun Yu, Can Yang, Siyan Wen, Yingchun Pan

**Affiliations:** 1 School of Philosophy and Law and Political Science, Shanghai Normal University, Shanghai, China; 2 School of Mental Health, Wenzhou Medical University, Wenzhou, China; 3 School of Public Administration, Guangdong University of Foreign Studies, Guangzhou, China; 4 School of Psychology and Cognitive Science, East China Normal University, Shanghai, China; 5 Faculty of Psychology, Southwest University, Chongqing, China; 6 College of Public Administration, Zhejiang University of Finance and Economics, Hangzhou, China

**Keywords:** adolescents’, community social capital, inter-generational transmission, psychological capital, social capital, spatial stratification

## Abstract

The inter-generational transmission of psychological capital plays a vital role in promoting urban adolescents’ development and mental health. However, prior research has predominantly focused on intra-family mechanisms while giving insufficient attention to the mediating role of social capital and the broader community context. Using an interdisciplinary perspective between psychology and sociology, this study examined how parental psychological capital influences adolescent psychological capital through social capital and explored the moderating effect of community type. This cross-sectional study collected survey data from 502 families in Shanghai, each comprising one high school student and both parents (total *N* = 1,506 participants). The results indicated that family (parental) psychological capital, social capital, and adolescent psychological capital were all significantly and positively correlated. Parental psychological capital was significantly and positively correlated with adolescent psychological capital, with social capital partially mediating this relationship. Community type significantly moderated the path from parental psychological capital to social capital, with the mediating effect being stronger in urban communities with high proximity to the downtown area compared to communities in suburban areas. These findings highlight the contextual dependence of the inter-generational transmission of psychological capital, underscore the critical role of social capital and community stratification in adolescent psychological development, and provide both theoretical insights and practical implications for enhancing family support and community-based interventions.

## 1 Introduction

### 1.1 Background

Psychological capital, representing positive psychological resources, is an important factor for adolescents’ development in urban areas. Psychological capital manifests as positive psychological states covering four primary dimensions: self-efficacy (i.e., having confidence and exerting effort to achieve success when faced with challenging tasks), optimism (i.e., positively attributing current and future success), hope (i.e., persistently pursuing goals, adjusting pathways to achieve success), and resilience (i.e., persisting and quickly recovering in adversity, surpassing challenges to achieve success) (1). Research has shown that, as a psychological resource, positive psychological capital is closely associated with reduced loneliness (2), decreased depression (3), and increased happiness (4).

At the psychological resource level, psychological capital may be transmitted between parents and offspring (i.e., inter-generational transmission; (5, 6)). Previous research has found that hope and resilience can be passed down across generations (7–9). Furthermore, compared to adolescents, the values of young adults are more similar to those of their parents (6). At the social resource level, studies have indicated that community social capital, defined as trust, reciprocity norms, and social networks embedded in neighborhood contexts beyond the immediate family unit (10)—may play a mediating role (11, 12). Associated with positive psychological states, parents may establish and maintain strong social networks within the community (4, 13–16), and adolescents may be able to benefit from these networks to cultivate positive psychological capital (16–22). Simultaneously, from the spatial stratification has been seen as a crucial moderator regarding environmental resources (11). As an objective residential environment, spatial stratification facilitates the formation and development of social resources through the distribution of high-quality resources, possibly enhancing the positive relationship between parental psychological capital and community social capital (23–27).

Although family and parents play a critical role in adolescent psychological development, previous research has lacked comprehensive investigation of the inter-generational transmission of psychological capital (28, 29). In addition, research related to psychological capital has often treated such characteristics as an independent factor with less consideration for the roles of social community and spatial environmental factors. As mentioned above, this triple resource combination (psychological, social, and environmental) is important to understand the processes promoting or hindering psychological intergeneration transmission across generations. Therefore, the current study conducted a questionnaire survey on the inter-generational transmission of psychological capital, including both community social capital as a social resource and spatial stratification as an environmental resource.

This study chose Shanghai as the study site as it represents the “urban explosion” in China, one of the fastest growing “newly industrialized countries (NIC).” In NIC, people have moved from rural regions to mega-cities for better employment opportunities and career development (30). As cities increase in population, residential spatial stratification based on neighborhood types and proximity to the downtown area may also emerge (31–34). Such spatial stratification has shown strong connections with both positive psychological traits and social capital (24, 27). While the theoretical mechanisms of intergenerational transmission are expected to be generalizable, certain aspects may be amplified in Chinese urban contexts. Specifically, collectivist cultural orientations may strengthen both family transmission and community mediation, while China’s rapid urbanization creates pronounced spatial stratification effects. However, the fundamental processes whereby parental resources relate to offspring outcomes through community mechanisms represent theoretically generalizable principles. Thus, we aim to explore the mediating role of community psychological capital and the moderating effect of spatial stratification in this process of psychological capital transmission. Ultimately, this study will provide theoretical and practical foundations for family educational interventions and the cultivation of positive psychological resources in an urban context.

### 1.2 Inter-generational transmission of psychological capital

The inter-generational transmission of psychological capital refers to positive psychological states, such as self-efficacy, hope, optimism, and resilience passed down from generation to generation (35, 36). inter-generational correlation is an indicator of inter-generational transmission (6). The greater the similarity between offspring and parental psychological capital, the stronger the inter-generational transmission effect. According to the social learning theory, parents play significant roles within the family, allowing offspring to observe and learn positive psychological states. Psychological capital is transmitted through parental role modeling and offspring’s observational learning (5). Therefore, psychological capital may exhibit a significant inter-generational correlation.

Empirical evidence suggests that positive psychological states may be transmitted across generations. For example, Huang et al. (37) found that individuals whose parents hold more positive evaluations of their own abilities and values tend to exhibit a more positive self-perception and greater self-affirmation. Wang (8) conducted a latent profile analysis on the joint characteristics of personality and intelligence, discovering that when parents exhibit high levels in the “resilience” trait combination, their offspring also demonstrate greater resilience in adversity. Similarly, questionnaire surveys and qualitative studies alike have highlighted the inter-generational transmission effect of hope between generations (7, 9). In terms of goal attainment strategies, adolescents with increased hope have reported their parents providing them with more guidance and encouragement, thus educating them to perceive obstacles as challenges. Furthermore, Barni et al. (6) revealed that families with children who are young adults demonstrate a greater similarity in values between parents and offspring compared to families with adolescent children. This may be due to efforts by parents to instill values during their offspring’s developmental process. Thus, based on empirical research on the inter-generational transmission of various positive psychological states, we inferred that psychological capital may be passed down through generations.

In conclusion, we hypothesized that a positive correlation may exist between parental psychological capital and adolescent psychological capital (Hypothesis 1, H1).

### 1.3 The mediating role of community social capital

Community social capital encompasses the social resources collectively possessed by community members, such as community social networks, belongingness, and trust (10, 25, 38), which contribute to the enhancement of psychological health (4). Drawing upon the conservation of resources theory, individuals constantly preserve and acquire various types of resources; to safeguard and sustain social relationships, people invest existing resources to acquire new resources (12). Consequently, higher levels of parental psychological capital are associated with more extensive community social capital. Research has indicated that people with abundant psychological capital demonstrate a higher ability to cultivate and maintain strong social connections. They are more likely to receive support from others and engage in social activities, thereby enhancing social capital (13). Hidayat et al. (14) also discovered that individuals with higher levels of positive psychological capital exhibit stronger interpersonal skills. They establish friendly relationships, actively expand their social networks, and tend to help and support others, and this tendency has been observed in a variety of circumstances. Luo et al. (15) suggested that hotel employees with ample psychological capital tend to trust interpersonal communication, enabling them to gain trust from both the organization and colleagues and access additional social resources. A survey conducted on unemployed youth revealed that graduates with high levels of self-efficacy, hope, optimism, and resilience accumulate social capital through various means to enhance employability (16). During the COVID-19 period, residents with a positive attitude and greater confidence in the future exhibited deeper trust in their neighbors and generated closer connections with each other (4). Hence, there may be a positive relationship between parental psychological capital and community social capital.

The ecological systems theory posits that the microsystems in which people directly engage (e.g., communities) play a vital role in psychological development (11). Correspondingly, the acquisition and utilization of community resources may be associated with psychological capital development. The richer the community social capital acquired by parents, the more supportive resources their children may have in the community, potentially resulting in a more positive psychological state for the offspring. The positive outcomes of social support are typically psychological (i.e., individuals can develop positive emotions and cognitions; (21)). Research has demonstrated that college students who perceive rich social capital tend to show higher self-efficacy, optimism, resilience, and hope (20). Paiva et al. (22) found that adolescent social capital is formed through interactions with parents, peers, and teachers; the more social support adolescents receive from important individuals in their lives, the higher their levels of psychological capital (2). Additionally, people within a community who possess trust and share a common vision are more likely to believe in their ability to achieve goals and accomplish tasks (17, 19). For instance, a study by Hsu and Chang (18) found that in gaming communities, players with stronger social connections also possess more psychological capital. Thus, there may be a positive relationship between community social capital and offspring’s psychological capital.

In summary, community social capital may play a mediating role in the inter-generational transmission of psychological capital. Specifically, the more positive the parental psychological capital, the richer their community social capital, and the more positive the psychological capital of the offspring (H2).

### 1.4 The moderating role of spatial stratification

A systematic review revealed a significant relationship between spatial stratification, environment, and social capital (23). According to the ecological systems theory, the interaction between individuals and their environment significantly impacts both psychological states and environmental resources (11). When parents possess positive psychological capital, spatial stratification may serve as an environmental resource that associated with the development of community social capital. Wang et al. (25) emphasized the importance of spatial factors on social relationships and community cohesion. Specifically, residing in areas with moderate population density, high bus stop density, and accessibility to commercial amenities has been shown to increase interaction and deepen trust among community members. Similarly, Won and Lee (26) confirmed that both perceived and objective residential environments are closely associated with social capital. The more comfortable living experiences are (e.g., well-equipped facilities, sufficient green spaces, and convenient access to public transportation and supermarkets), the more residents trust their neighbors and are willing to interact with them. At the same time, residents are more likely to receive help from their neighbors. Moreover, Zhang et al. (27) revealed that the interaction between positive personality traits and spatial stratification promotes positive affect in China.

In the Chinese context, types of housing and location of residential neighborhoods may both illustrate two axes of spatial stratification (33, 34, 39). Regarding housing types, residents living in commercial housing communities have been shown to enjoy increased access to social resources compared to their counterparts living in resettled housing communities (34). Resettled housing communities are often relocated from existing traditional “soviet-style” neighborhoods in urban areas, which are usually distributed and built by local governments before the 1998 Chinese housing market reform (*Hukou*) (40). Compared with commercial housing, resettled housing communities are usually of a lower quality and do not share equal property rights, such as mortgage loans and transaction restrictions (41). As a result, resettled housing communities are viewed as “cheaper” and are thus ranked lower on the hierarchy of urban spatial strata than commercial housing (25).

Furthermore, large metropolitan areas in newly industrialized countries follow traditional urban development patterns, where public social resources are mainly concentrated in the downtown area (31, 32, 42, 43). In the case of Shanghai, research often categorizes the city spaces into four zones based on three transportation road loop “rings” across the city, indicating their proximity toward the downtown area: within the “inner ring;” between the “inner ring” and “middle ring;” between the “middle ring” and “outer ring;” and outside the “outer ring” (44, 45). As the general population and commercial hub are dominantly located within the downtown area, the density of public transportation and public services gradually decreases from the “inner ring” toward outside the “outer ring” ((44, 46)). Consequently, these road loop “rings” become the agent for Shanghai urban spatial stratification: Housing prices exhibit a notable decline as one moves from the inner ring to the outer ring (45).

All in all, individuals from communities with higher levels of spatial stratification, who typically have better economic conditions, exhibit different emotional patterns compared to those from lower spatial stratifications. In the presence of abundant environmental resources, their optimistic personality traits are more highly related to positive affect. Moreover, positive affect is positively correlated with social capital (24). The moderating effects of spatial stratification may operate through multiple interconnected mechanisms. First, high-spatial-stratification communities (e.g., commercial housing in central urban areas) provide superior resource acquisition opportunities through concentrated infrastructure, services, and social interaction spaces, facilitating parents’ conversion of psychological capital into accessible community networks. Second, these communities may foster stronger normative expectations around community engagement and reciprocity, creating social environments where psychological resources are more readily translated into social capital. Third, spatial proximity in well-resourced neighborhoods reduces transaction costs for social participation, enabling parents with high psychological capital to more efficiently build and maintain community ties. Conversely, lower-stratification communities face structural constraints—limited facilities, dispersed populations, longer commuting distances—that impede the conversion of individual psychological resources into collective social capital, regardless of parental psychological capital levels. These mechanisms suggest that spatial stratification functions not merely as a descriptive characteristic but as a systematic moderator shaping resource conversion efficiency. Thus, we inferred that spatial stratification may enhance the relationship between parental psychological capital and community social capital. In other words, spatial stratification was expected to moderate the relationship between the two (H3).

### 1.5 The present study

**Figure 1:**
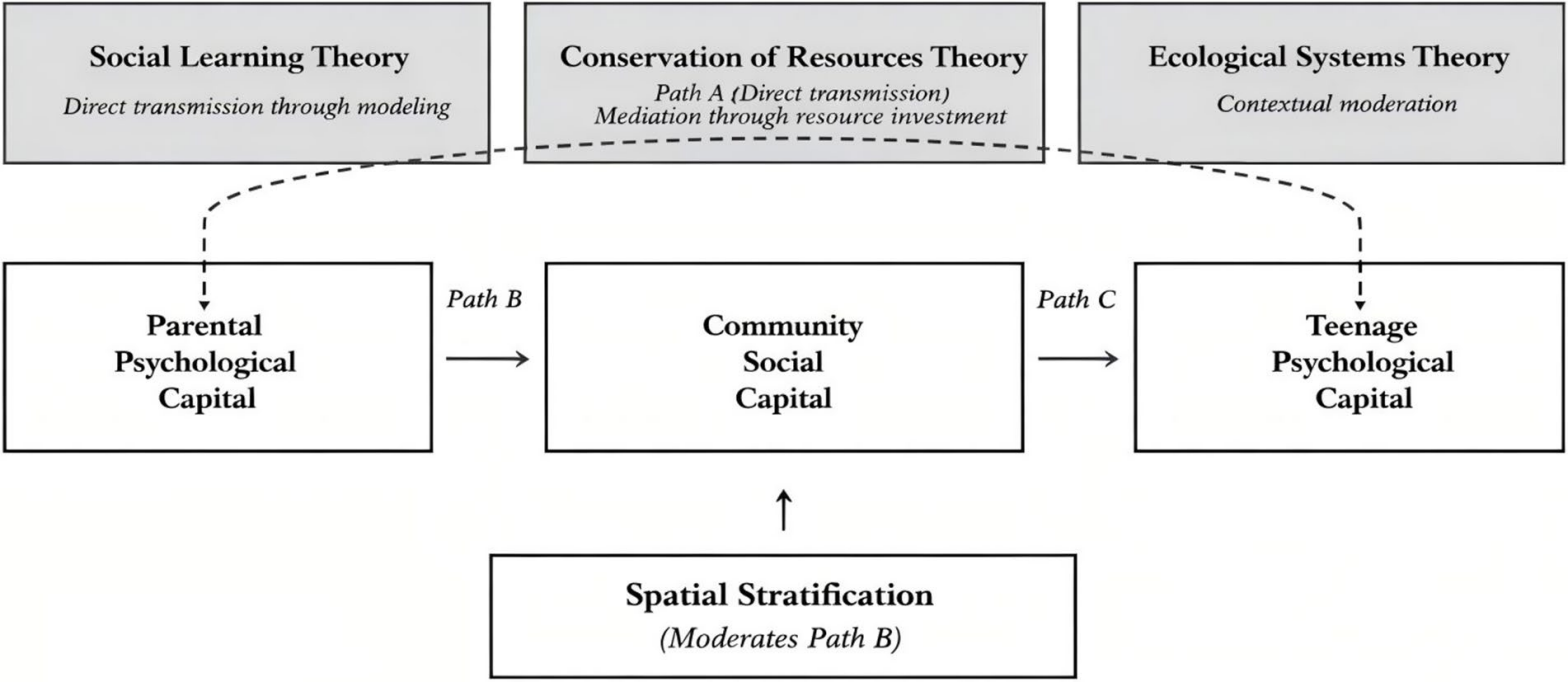
Theoretical integration framework.

This study integrates three complementary theoretical perspectives to examine intergenerational psychological capital transmission (see Figure 1). Social learning theory (5) explains the direct transmission pathway whereby teenagers acquire psychological capital through observational learning and modeling of parental behaviors. Conservation of resources theory (12) explicates the mediating mechanism: parents with abundant psychological resources invest these resources to acquire community social capital, which subsequently becomes available to support teenage development. Ecological systems theory (11) provides the contextual framework, positing that the effectiveness of family and community resource processes depends on environmental contexts—specifically, spatial stratification as an exosystem-level factor that moderates resource accessibility and utilization. Together, these theories form an integrated framework where direct parent–child transmission (social learning) operates alongside community-mediated pathways (resource conservation), with both processes contingent upon spatial-environmental contexts (ecological systems). We employed a cross-sectional survey in Shanghai to examine these integrated pathways, testing the mediating role of community social capital and the moderating role of spatial stratification in the relationship between parental and teenage psychological capital.

## 2 Methods

### 2.1 Participants

To recruit families with high school students, we used a purposive, school-based sampling strategy and collected data from eight Shanghai high schools selected to balance both regional distribution and educational tier: two schools (one key and one general public high school) were included from each of the city’s four ring-road zones (within the Inner Ring, Inner–Middle Ring, Middle–Outer Ring, and beyond the Outer Ring). With the coordination of school moral education offices, Grade 10–12 students were invited through class-based recruitment; each participating student and both parents completed the questionnaires independently.

#### 2.1.1 Inclusion and exclusion criteria

Families were eligible if (a) the student was enrolled in Grades 10–12, (b) the father and mother co-resided with the student in the same household at the time of the survey, and (c) all respondents could complete the Chinese questionnaire independently. Families were excluded if (a) key variables were substantially missing or responses showed clearly invalid patterns (e.g., straight-lining), (b) either parent was not co-residing with the student, or (c) any respondent had language, cognitive, or other barriers preventing valid questionnaire completion. Co-residence status was collected in the family information section and used as an eligibility criterion.

A total of 823 family samples were initially collected; after applying the criteria above, 502 valid family samples were retained. The final dataset included 1,506 participants (502 students and their two parents). All participating parents reported having at least a bachelor’s degree, which likely reflects the educational profile of families served by the participating schools and the voluntary nature of parent participation in school-based surveys. This study was conducted in accordance with ethical guidelines for research involving minors. Both parental informed consent and adolescent assent were obtained prior to data collection. To ensure confidentiality and data protection, all responses were anonymized using unique identification codes, and data were stored securely with access restricted to authorized research personnel only. Participant information is shown in Table 1.

**Table 1:** Sample characteristics (*N* = 502 families; 1,506 individuals).

Characteristic	Category/Value	*n* (%)/Mean ± SD
Adolescents		
Grade	Grade 10	170 (33.9)
	Grade 11	161 (32.1)
	Grade 12	171 (34.1)
Gender	Male	259 (51.6)
	Female	243 (48.4)
Age (years)	Mean ± SD (range)	16.21 ± 0.93 (15–18)
Parents		
Father age (years)	Mean ± SD (range)	41.85 ± 2.76 (38–49)
Mother age (years)	Mean ± SD (range)	41.09 ± 2.59 (38–49)
Father education	Bachelor/Associate	301 (60.0)
	Master	119 (23.7)
	Doctoral	82 (16.3)
Mother education	Bachelor/Associate	344 (68.5)
	Master	123 (24.5)
	Doctoral	35 (7.0)
Household/neighborhood		
Ring-road residential location	Within Inner Ring	129 (25.7)
	Between Inner to Middle ring	115 (22.9)
	Between Middle to Outer ring	140 (27.9)
	Outside of Outer Ring	118 (23.5)
Housing type	Commercial	250 (49.8)
	Resettled (non-commercial)	252 (50.2)

Values are *n* (%) unless otherwise indicated. Age is reported in years.

### 2.2 Research design

#### 2.2.1 Family (parental) psychological capital

The “Positive Psychological Capital Questionnaire for China,” developed from the previous study (67), was used to measure the psychological capital levels of both fathers and mothers in Shanghai. This scale includes four dimensions: self-efficacy, hope, optimism, and resilience, with a total of 26 questions. It uses a Likert 7-point scoring system (1 = “strongly disagree” to 7 = “strongly agree”), with higher scores indicating higher levels of individual psychological capital. This scale has good reliability and validity and is widely used in Chinese environments. In this study, Cronbach’s alpha coefficients of the father version and mother version of the questionnaires were 0.90 and 0.89, respectively, indicating good reliability. As the family system theory holds that the family is a dynamic emotional unit, the psychological resources of both the father and the mother would influence each other and jointly constitute the overall psychological environment of the family (47). Based on this, this study added the psychological capital scores of the fathers and mothers to construct an integrated indicator of “family (parental) psychological capital.” This operation aims to measure the overall level of psychological advantage resources from both parents, and its rationality is based on the following two points: Firstly, the psychological capital scores of the father and mother are significantly positively correlated (r = 0.72, *p* < 0.001), indicating a common trend of change between the two; secondly, the intra group consistency index is 0.82, further supporting the statistical proof of using the sum of parental data as family psychological capital. The higher the family psychological capital final score, the richer the overall positive psychological resources possessed by the family.

#### 2.2.2 Adolescent psychological capital

The similar “Positive Psychological Capital Questionnaire for China” was also used to measure the psychological capital level of the adolescents. This scale used four similar dimensions (self-efficacy, hope, optimism, and resilience) with 24 similar questions as the family (parental) psychological capital scale mentioned above, also ranked with a Likert 5-point scoring system. The Cronbach’s alpha coefficient of the adolescent psychological capital questionnaire was 0.90, indicating good reliability.

#### 2.2.3 Community social capital

This study adopted the Chinese Social Capital Measurement Indicators to assess community social capital (10). The scale covers seven dimensions, including local social networks, community belonging, community cohesion, non-local social interactions, reciprocity, and general trust, with a total of 29 items rated on a 5-point Likert scale (1 = strongly disagree to 5 = strongly agree). Higher scores indicate higher community social capital. In the present study, Cronbach’s alpha was 0.90, indicating good reliability. In the Chinese context, community social interactions are often organized at the household level, and family members’ community participation, social networks, and trust tend to be shared and interdependent (48, 49). Accordingly, each family designated either the father or the mother to complete the community social capital questionnaire as the household informant, consistent with prior practice (50).

#### 2.2.4 Spatial stratification

The spatial stratification level was measured and operationalized with two methods: type of housing for participating families, and the residential location of participants’ homes (based on the transportation loop). These methods follow the universal paradigm for studying the spatial stratification of Chinese cities (25, 51), aiming to explore the multidimensional social class boundaries of Chinese cities. In this study, housing type and ring-based residential location were treated as two distinct indicators of spatial stratification and were analyzed independently.

In terms of housing types, the participants were divided into two types of housing. Commercial housing owners (coded as 1) refer to residents who obtained commercial housing through market purchases. This group usually has complete market property rights, and their housing symbolizes higher economic capital and social status (25). Non-commodity housing owners (coded as 0) refer to residents who obtain housing through non-market channels, such as policy-based resettlement, and their housing acquisition methods reflect institutional arrangements rather than market choices. This binary division effectively identifies two social status groups, with commercial housing as a higher level of spatial stratification.

In terms of the ring-road residential location, this study draws on the core-periphery theory from urban geography to categorize urban residents into four spatial zones: within the inner ring (coded as 4), inner ring to middle ring (coded as 3), middle ring to outer ring (coded as 2), and outside the outer ring (coded as 1). This gradient coding captures locational advantage, with higher values indicating better transportation convenience, richer spatial resources, and greater access to infrastructure, opportunities, and social status. By employing this location-based classification, this study aims to empirically test how multi-dimensional spatial stratification influences residents’ social psychology and behaviors (44).

### 2.3 Data processing and statistical analysis

Data analysis was performed using SPSS 27.0. Harman’s single-factor test was used to evaluate common method bias, and Pearson correlation analyses were conducted to examine bivariate associations among the study variables. To test the hypotheses, mediation and moderated mediation were analyzed using the PROCESS macro. Specifically, Model 4 was used to test the mediation model, and Model 7 was used to test the first-stage moderated mediation model, following Hayes (52). Adolescent gender, grade, age, parental education, and parental age were included as covariates in all models. Indirect effects were tested using bias-corrected percentile bootstrapping with 5,000 resamples to generate 95% confidence intervals. All continuous variables were standardized prior to the analysis to reduce multicollinearity and facilitate interpretation of interaction terms.

## 3 Results

### 3.1 Common method deviation test

To reduce potential common-method bias during data collection, the survey was administered anonymously (non-identifiable responses), included some reverse-worded items, and collected key variables from multiple informants (parents and adolescents), thereby reducing single-source bias. In the analysis stage, Harman’s single-factor test was conducted. The first unrotated factor accounted for 17% of the total variance, below the commonly used 40% criterion, suggesting that common-method variance was unlikely to be a major concern in the present study.

### 3.2 Descriptive statistics and correlation analysis

Prior to the main analysis, the distribution of the data was examined. The skewness values for the study variables ranged from −0.43 to 0.33, and the kurtosis values ranged from −1.32 to −0.94. These statistics indicate that the data are normally distributed, as they fall well within the acceptable range of ±2 (53). Additionally, an inspection of standardized z-scores revealed no outliers exceeding the ±3.29 threshold. Given the large sample size (*N* = 502) and the normal distribution of the data, the dataset was deemed suitable for the subsequent mediation and moderation analyses.

Table 2 lists the mean, standard deviation, and correlation matrix of each variable. The results showed significant positive correlations among all variables.

**Table 2:** Descriptive statistics and correlation analysis results.

Variable	M ± SD	1	2	3
1. Family psychological capital	236.03 ± 70.15	1		
2. Community social capital	178.76 ± 108.85	0.61^**^	1	
3. Adolescent psychological capital	120.62 ± 39.60	0.77^**^	0.66^**^	1

^*^p < 0.05, ^**^p < 0.01, ^***^p < 0.001.

Partial correlation analyses were conducted, controlling for adolescent grade, gender, and age, as well as parental age and education. The associations remained significant (family psychological capital with adolescent psychological capital: pr = 0.77, *p* < 0.001; family psychological capital with community social capital: pr = 0.61, *p* < 0.001; community social capital with adolescent psychological capital: pr = 0.65, *p* < 0.001), indicating robust relationships after adjustment.

### 3.3 Mediation effect test

Using Hayes’ PROCESS macro (Model 4), we tested the mediating role of community social capital while controlling for adolescent gender, grade, and age, as well as parental education and parental age. As shown in Table 3, family psychological capital had a significant total effect on adolescents’ psychological capital (*β* = 0.77, SE = 0.03, *t* = 27.02, 95% CI [0.72, 0.83]) and remained significant after accounting for the mediator (direct effect: β = 0.59, SE = 0.03, *t* = 17.66, 95% CI [0.53, 0.66]). Bias-corrected bootstrap analyses indicated a significant indirect effect through community social capital (ab = 0.18, SE = 0.04, 95% CI [0.10, 0.26]), accounting for 23.38% of the total effect, supporting partial mediation.

**Table 3:** Mediation effects of community social capital on the relationship between paternal psychological capital and adolescent psychological capital.

Effect	Point estimate	SE	*t*	Bias-corrected 95% CI	
				LL	UL
Total effect (c)	0.77	0.03	27.02^***^	0.72	0.83
Indirect effect (ab)	0.18	0.04	–	0.10	0.26
Direct effect (c′)	0.59	0.03	17.66^***^	0.53	0.66
Proportion mediated	23.38%				

^*^p < 0.05, ^**^p < 0.01, ^***^p < 0.001.

Models controlled for adolescent gender, grade, age, parental education, and parental age.

### 3.4 Moderated mediation effect test

#### 3.4.1 The moderating effect of spatial stratification (housing type) on the mediating effect of community social capital

Using Hayes’ PROCESS Model 7, we tested the moderating role of housing type while controlling for adolescent gender, grade, and age, as well as parental education and parental age. As shown in Table 4, parental psychological capital positively predicted community social capital (*β* = 0.14, *p* < 0.05, 95% CI [0.03, 0.26]), and housing type also positively predicted community social capital (*β* = 0.98, *p* < 0.001, 95% CI [0.82, 1.16]). Importantly, the interaction between parental psychological capital and housing type was significant (*β* = 0.26, *p* < 0.01, 95% CI [0.09, 0.43]), indicating that housing type moderated the association between parental psychological capital and community social capital. Community social capital, in turn, significantly predicted adolescent psychological capital (*β* = 0.30, *p* < 0.001, 95% CI [0.23, 0.36]), and the direct effect of parental psychological capital on adolescent psychological capital remained significant (*β* = 0.59, *p* < 0.001, 95% CI [0.53, 0.66]).

**Table 4:** Moderated mediation analysis: housing type as moderator.

Variables	Model 1: Community social capital (M)				Model 2: Adolescent psychological capital (Y)			
	*β*	*SE*	*t*	95% CI	*β*	*SE*	*t*	95% CI
Gender	0.12	0.06	1.90	[−0.004, 0.24]	0.003	0.05	0.07	[−0.10, 0.11]
Grade	0.05	0.10	0.53	[−0.15, 0.26]	−0.18	0.09	−2.04^*^	[−0.35, −0.01]
Adolescent age (years)	0.02	0.08	0.22	[−0.14, 0.17]	0.06	0.07	0.93	[−0.07, 0.19]
Father’s education	−0.12	0.17	−0.66	[−0.46, 0.23]	−0.29	0.15	−1.96	[−0.58, 0.001]
Mother’s education	0.03	0.09	0.37	[−0.14, 0.21]	−0.03	0.07	−0.37	[−0.18, 0.12]
Father’s age (years)	0.02	0.04	0.50	[−0.06, 0.10]	0.06	0.03	1.89	[−0.002, 0.13]
Mother’s age (years)	0.004	0.03	0.12	[−0.06, 0.07]	0.03	0.03	0.97	[−0.03, 0.08]
Parental psychological capital (X)	0.14	0.06	2.46^*^	[0.03, 0.26]	0.59	0.03	17.66^***^	[0.53, 0.66]
Housing type (W)	0.98	0.09	11.44^***^	[0.82, 1.16]	–	–	–	–
X × W (interaction)	0.26	0.09	2.96^**^	[0.09, 0.43]	–	–	–	–
Community social capital (M)					0.30	0.03	8.80^***^	[0.23, 0.36]
*R* ^2^	0.52				0.66			
*F*	53.72^***^				104.29^***^			

^*^p < 0.05, ^**^p < 0.01, ^***^p < 0.001.

Simple slope analyses indicated that when housing type was non-commercial (resettled) housing (W = 0), the conditional effect of parental psychological capital on community social capital was 0.14 (SE = 0.06, *t* = 2.46, *p* < 0.05, 95% CI [0.03, 0.26]). When housing type was commercial housing (W = 1), the conditional effect increased to 0.40 (SE = 0.07, *t* = 6.21, *p* < 0.001, 95% CI [0.27, 0.53]). These results suggest that the positive association between parental psychological capital and community social capital was stronger among families living in commercial housing.

Further, we used bias-corrected bootstrapping with 5,000 resamples to estimate the conditional indirect effects. The indirect effect of parental psychological capital on adolescent psychological capital via community social capital was significant both for families living in non-commercial (resettled) housing (W = 0; indirect effect = 0.04, BootSE = 0.02, 95% BootCI [0.01, 0.09]) and for those living in commercial housing (W = 1; indirect effect = 0.12, BootSE = 0.04, 95% BootCI [0.05, 0.19]), as neither confidence interval included zero. The index of moderated mediation was 0.08 (BootSE = 0.04, 95% BootCI [−0.004, 0.16]). Because the confidence interval slightly crossed zero, the moderated mediation effect did not reach significance at the 95% confidence level, suggesting only a trend toward a stronger indirect effect in commercial-housing contexts.

#### 3.4.2 The moderating effect of spatial stratification (ring-road residential location) on the mediating effect of community social capital

Using Hayes’ PROCESS Model 7, we tested the moderating role of ring-road residential location while controlling for adolescent gender, grade, and age, as well as parental education and parental age. As shown in Table 5, parental psychological capital positively predicted community social capital (β = 0.67, *p* < 0.001, 95% CI [0.60, 0.75]), whereas ring-road residential location negatively predicted community social capital (β = −0.12, *p* < 0.01, 95% CI [−0.19, −0.04]). Importantly, the interaction between parental psychological capital and ring-road residential location was significant (β = 0.11, *p* < 0.01, 95% CI [0.04, 0.18]), indicating that ring-road residential location moderated the association between parental psychological capital and community social capital. Community social capital, in turn, significantly predicted adolescent psychological capital (β = 0.30, *p* < 0.001, 95% CI [0.23, 0.36]), and the direct effect of parental psychological capital on adolescent psychological capital remained significant (β = 0.59, *p* < 0.001, 95% CI [0.53, 0.66]).

**Table 5:** Moderated mediation analysis: Ring-road residential location as moderator.

Variables	Model 1: Community social capital (M)				Model 2: Adolescent psychological capital (Y)			
	*β*	*SE*	*t*	95% CI	*β*	*SE*	*t*	95% CI
1. Gender	0.09	0.07	1.32	[−0.05, 0.23]	0.003	0.05	0.07	[−0.10, 0.11]
2. Grade	−0.05	0.11	−0.45	[−0.28, 0.17]	−0.18	0.09	−2.04^*^	[−0.35, −0.01]
3. Adolescent age (years)	0.05	0.09	0.52	[−0.13, 0.22]	0.06	0.07	0.93	[−0.07, 0.19]
4. Father’s education	−0.26	0.20	−1.34	[−0.65, 0.12]	−0.29	0.15	−1.96	[−0.58, 0.001]
5. Mother’s education	0.06	0.10	0.60	[−0.14, 0.26]	−0.03	0.07	−0.37	[−0.18, 0.12]
6. Father’s age (years)	0.05	0.04	1.22	[−0.03, 0.14]	0.06	0.03	1.89	[−0.002, 0.13]
7. Mother’s age (years)	0.01	0.04	0.30	[−0.06, 0.08]	0.03	0.03	0.97	[−0.03, 0.08]
Parental psychological capital (X)	0.67	0.04	17.46^***^	[0.60, 0.75]	0.59	0.03	17.66^***^	[0.53, 0.66]
Ring-road residential location (W)	−0.12	0.04	−3.05^**^	[−0.19, −0.04]	–	–	–	–
X × W (interaction)	0.11	0.04	3.12^**^	[0.04, 0.18]	–	–	–	–
Community social capital (M)					0.30	0.03	8.80^***^	[0.23, 0.36]
*R^2^*	0.40				0.66			
*F*	32.92^***^				104.29^***^			

^*^p < 0.05, ^**^p < 0.01, ^***^p < 0.001.

Simple slope analyses showed that the conditional effect of parental psychological capital on community social capital was 0.56 (SE = 0.05, *t* = 12.35, *p* < 0.001, 95% CI [0.47, 0.65]) at a more peripheral ring-road location (W = −1 SD), 0.67 (SE = 0.04, *t* = 17.46, *p* < 0.001, 95% CI [0.60, 0.75]) at the mean level (W = M), and 0.78 (SE = 0.06, *t* = 13.21, *p* < 0.001, 95% CI [0.67, 0.90]) at a more central ring-road location (W = +1 SD). These results suggest that the positive association between parental psychological capital and community social capital was stronger in more central locations (i.e., closer to the Inner Ring).

Further, we used bias-corrected bootstrapping with 5,000 resamples to estimate the conditional indirect effects. The indirect effect of parental psychological capital on adolescent psychological capital via community social capital was significant at more peripheral locations (indirect effect = 0.17, Boot SE = 0.04, 95% Boot CI [0.09, 0.24]), at the mean level (indirect effect = 0.20, Boot SE = 0.04, 95% Boot CI [0.12, 0.28]), and at more central locations (indirect effect = 0.23, Boot SE = 0.05, 95% Boot CI [0.14, 0.34]), as none of the confidence intervals included zero. The index of moderated mediation was 0.03 (Boot SE = 0.02, 95% Boot CI [−0.001, 0.07]). Because the confidence interval slightly crossed zero, the moderated mediation effect did not reach significance at the 95% confidence level, suggesting only a trend toward a stronger indirect effect in more central ring-road locations.

#### 3.4.3 Robustness check and supplementary analysis

To validate the use of the aggregated family psychological capital score, we conducted sensitivity analyses separating paternal and maternal effects (see Supplementary Tables S1–S6). The mediation results were robust: both paternal and maternal psychological capital independently predicted community social capital and adolescent outcomes (Supplementary Tables S1, S2). However, the moderation results underscored the importance of the aggregated approach. For Housing Type, the moderation was significant for fathers (Supplementary Tables S3) but not mothers (Supplementary Tables S4). Notably, for Residential Location, the interaction was not significant for either parent individually (Supplementary Tables S5, S6) but was significant for the aggregated family score (Table 5). This suggests that the moderating influence of macro-spatial factors (like urban location) relies on the cumulative psychological resources of the entire household, supporting the theoretical decision to model family psychological capital as a collective construct. All in all, the results were robust and double checked.

## 4 Discussion

The main aim of this study was to examine the relationship between parents’ psychological capital and adolescents’ psychological capital for high school students in Shanghai. It evaluated the mediating role of community social capital and the moderating role of spatial stratification in this linkage. This study specifically investigated how community social capital is associated with a mechanistic pathway in the relationship between parents’ psychological resources and teenagers’ psychological capital, and how this entire transmission process is contingent upon a multi-dimensional spatial stratification (encompassing both the type of housing and the location of the participants’ home).

### 4.1 Inter-generational transmission of psychological capital

The results of this study indicated a significant positive correlation between family psychological capital and adolescent psychological capital, which was consistent with the hypothesis. This discovery confirmed the significant transmission effect of psychological capital between family generations, similar to most previous research findings (7–9, 37, 54, 55). Our study extends prior research by examining psychological capital as a composite construct rather than isolated dimensions, and the strength of this transmission in our Shanghai sample may reflect the particularly strong family interdependence characteristic of Chinese collectivist culture (56), which facilitates more extensive sharing of psychological resources compared to individualistic contexts.

Psychological capital, as a positive psychological state developed by individuals during their growth process, not only depends on personal experiences but is also largely influenced by family environment (11). As important figures in their children’s growth process, parents may transmit their psychological capital elements, such as optimism, resilience, self-efficacy, and hope, to their children through role modeling, emotional support, parenting styles, and other means (5). The mechanisms underlying this transmission involve teenagers observing and internalizing not only parental behaviors but also their cognitive schemas and attributional patterns, as demonstrated by Huang et al.’s (37) finding that parental self-evaluations shape offspring’s self-perception. In the context of collectivist culture in Chinese families, the transmission of psychological and behavioral patterns between generations is even more prominent, and the psychological resources of family members are more easily shared and diffused (56). Thus, there is a positive relationship between parental psychological capital and Chinese adolescents’ psychological capital.

### 4.2 The mediating role of community social capital

This study further found that community social capital plays an important mediating role in the inter-generational correlation of psychological capital, which was in line with the initial theoretical expectations of this study (11, 12). This discovery suggests that inter-generational transmission of positive psychological resources within families does not occur in isolation, implying that a broad community environment also plays an indirect role in this process. Parents with high psychological capital tend to actively participate in community interactions, establish reciprocal norms, and obtain social support, thereby enhancing the community social capital that the family can mobilize (4, 13–16). Moreover, rich community social capital further is associated with providing adolescents with a wider range of positive social interactions and external support resources, indirectly promoting their psychological capital growth (16–22, 57). Hence, community social capital plays a mediating role in the relationship between parental psychological capital and adolescent psychological capital. This mediating pathway differs from Western studies that primarily examine parent–child dyadic interactions in isolation (54). The prominence of community social capital in Chinese urban context may reflect the traditional emphasis on collective networks and neighborhood ties in East Asian societies. Such community resources are more actively leveraged for child development compared to individualistic Western contexts where families tend to rely more on nuclear family resources (10). Additionally, the partial rather than full mediation observed in our study suggests that direct parent–child transmission mechanisms (e.g., modeling, emotional support) remain important alongside community pathways, consistent with the dual-process model proposed by Bronfenbrenner’s ecological systems theory.

The trust, reciprocity norms, and social networks contained in community social capital constitute important contextual resources that affect individual development (10, 58). The results of this study indicate that families with higher parental psychological capital are more likely to actively construct and utilize social resources in the community. This process not only enhances the socialization level of the family itself but also provides a richer supportive environment for the psychological growth of adolescents (65, 66). The mediating effect exhibited by community social capital further elucidates that individual psychological development not only benefits from internal family resources but is also closely related to broader social connections and resource acquisition mechanisms (11, 12, 59). This discovery provides empirical support for understanding the synergistic effect of family and community in the inter-generational transmission of psychological capital, indicating the importance of social resources in the development of individual positive psychological qualities.

### 4.3 The moderating role of spatial stratification

In the indirect pathway where parental psychological capital was positively related to adolescent psychological capital through community social capital, spatial stratification moderated the first pathway. This fully meets the initial theoretical expectations of our study (11). Housing types (commercial housing and non-commercial housing) significantly enhanced the positive relationship of parental psychological capital and community social capital. This research has shown that compared to non-commercial housing residents, parents living in commercial housing possess more community social capital and which may be associated with nurturing their children’s psychological capital. This may be because commercial housing communities typically have higher quality community resources, more complete infrastructure, and a more active community participation culture (23). Results revealed the effect of differences in resource accessibility and social interaction opportunities among communities with different types of housing on the inter-generational transmission of psychological capital (60). Commercial housing communities are associated with more favorable environmental conditions for parents with high psychological capital to construct and utilize community social capital (25, 26, 61). In contrast, the function of non-commodity housing communities is relatively weak. Therefore, housing type plays a moderating role in the relationship between parental psychological capital and community social capital. This moderating effect is particularly salient in the Chinese context, where the distinction between commercial and non-commercial housing represents not only economic differences but also institutional boundaries created by China’s housing reform (25), potentially creating sharper resource disparities than typical Western residential segregation.

In terms of the residential location dimension, families in the inner ring exhibited a significantly stronger mediating effect than those in the middle ring and outer ring. This could be attributed to convenient transportation and the central urban location, which boost both the frequency and quality of interactions among community residents, thereby increasing the density of community social networks and strengthening reciprocal norms (62). Additionally, good transportation expands residents’ social activity space while reducing the space–time cost of community participation. These factors create more favorable conditions for converting parental psychological capital into community social capital (63), which may in turn be associated with the development of teenagers’ psychological capital. In conclusion, residential location dimensions also serve a moderating role in the relationship between parental psychological capital and community social capital (64). This gradient effect aligns with Shanghai’s monocentric urban structure, where resources concentrate in central areas (31). It suggests that spatial effects may be particularly pronounced in rapidly urbanizing Asian mega-cities.

### 4.4 Implications

Our study has advanced understandings of the intergenerational transmission of psychological capital by integrating the family, community, and spatial dimensions of the phenomenon. It has also demonstrated that psychological resources operate through community networks and that the process is contingent upon spatial contexts. In turn, the findings offer actionable implications for community-based interventions tailored to different spatially stratified communities in Shanghai.

For commercial housing communities in central areas of Shanghai, interventions could optimize existing resources through structured parent–child–community pathways, including community-organized psychological capital workshops and family volunteer programs that model resilience and goal-setting behaviors.

For the city’s noncommercial housing and suburban communities, which face especially significant challenges in building community social capital, interventions should prioritize several key strategies. First, accessible community centers should be established to facilitate social network building among residents. Second, government-subsidized programs should be implemented to improve community facilities and create spaces for social interaction. Third, mobile outreach services that bring psychological capital-building activities to underserved neighborhoods should be developed. Fourth and finally, inter-community exchange programs that connect families across spatial divides should be created.

At the family level, parent education programs could provide concrete strategies that include training on how to leverage community resources, peer support groups for sharing community engagement strategies, and workshops on cultivating hope, resilience, and optimism in daily interactions.

From the perspective of urban governance, Shanghai should also consider several policy directions. First, differential resource allocation should be considered because it favors spatially disadvantaged areas. Second, infrastructure improvements could be made to reduce spatial gradients in the accessibility of community services. Third, monitoring systems could be used to track disparities in social capital.

All those context-specific interventions acknowledge that spatially stratified urban environments require tailored approaches that address both family psychological resources and community infrastructure to effectively support adolescent development across diverse residential contexts.

### 4.5 Limitations

Despite its contributions, our study had several limitations. First, the cross-sectional design limited our ability to establish causal directionality, because alternative sequences (e.g., adolescents influencing parents) or reciprocal effects over time could not be ruled out. Future research should employ longitudinal or experimental designs to better establish causal precedence. Second, the sample was drawn solely from Shanghai and, due to purposive school-based recruitment, was socioeconomically advantaged (e.g., parents held at least a bachelor’s degree) and restricted to co-residing two-parent households. Those circumstances may limit the generalizability of the findings to more disadvantaged families. Third, parental psychological capital was modeled as a single family-level construct; although that conceptualization captures the overall household psychological resource climate, it also assumes equal parental weighting and may mask parent-specific effects or discrepancies. Fourth, community social capital was reported by one parent, which may have introduced informant bias and measurement error. Last, household income and community-level indicators (e.g., population density) were not assessed and therefore could not be controlled, and residual common-method bias may therefore remain given the reliance on self-report measures. Throughout our study, we adhered to ethical guidelines for research involving minors, obtained consent from parents and adolescents, and anonymized and securely stored all data to protect participants’ confidentiality.

## 5 Conclusion

In conclusion, family (parental) psychological capital is significantly and positively correlated with adolescent psychological capital, with community social capital playing a partially mediating role in this relationship. Furthermore, spatial stratification moderates the first stage of this mediating pathway—specifically, the relationship between family psychological capital and community social capital. This moderating effect is manifested in two dimensions: Both the type of housing (commercial housing vs. non-commodity housing) and the location of the participants’ home significantly enhance this relationship. That is, in communities characterized by commercial housing and an urban location with higher transportation convenience, the positive effect of family psychological capital on community social capital is stronger, thereby strengthening the entire indirect pathway of inter-generational transmission of psychological capital. These results collectively demonstrate that the inter-generational transmission of psychological capital is not only influenced by internal family resources but is also shaped by the broader socio-spatial environment.

## Data Availability

The raw data supporting the conclusions of this article will be made available by the authors, without undue reservation.
