# Supplementary material for: Inter-generational transmission of psychological capital for adolescents: the mediating role of community social capital and the moderating role of spatial stratification
Source: Front Public Health. 2026 Feb 18;14:1728505. doi: 10.3389/fpubh.2026.1728505 (PMC12959244; doi:10.3389/fpubh.2026.1728505)
Supplement: Supplementary file 1 [file Table_1.pdf]

Table S1. Mediation effects of community social capital on the relationship between Father's psychological capital and adolescent psychological capital.

Effect	Point estimate	SE	*t*	Bias-corrected 95% CI	
				LL	UL
Total effect (c)	0.70	0.03	21.97^{***}	0.63	0.76
Indirect effect (ab)	0.21	0.04	-	0.15	0.29
Direct effect (c′)	0.49	0.03	14.11^{***}	0.42	0.55
Proportion mediated	30%				

Note. Models controlled for adolescent gender, grade, age, parental education, and parental age.

Table S2. Mediation effects of community social capital on the relationship between Mother's psychological capital and adolescent psychological capital.

Effect	Point estimate	SE	*t*	Bias-corrected 95% CI	
				LL	UL
Total effect (c)	0.71	0.03	22.55^{***}	0.65	0.77
Indirect effect (ab)	0.21	0.04	-	0.15	0.28
Direct effect (c′)	0.50	0.03	14.77^{***}	0.44	0.57
Proportion mediated	29.58%				

Note. Models controlled for adolescent gender, grade, age, parental education, and parental age.

Table S3. Moderated mediation analysis: Housing type as the moderator for Father's psychological capital.

Variables	Model 1: Community social capital (M)				Model 2: Adolescent psychological capital (Y)			
	β	*SE*	*t*	95% CI	*β*	*SE*	*t*	95% CI
Gender	0.13	0.06	2.07^{*}	[0.01, 0.25]	0.01	0.06	0.13	[-0.11, 0.12]
Grade	0.09	0.10	0.93	[-0.10, 0.28]	-0.13	0.09	-1.49	[-0.30, 0.04]
Adolescent age (years)	0.02	0.08	0.20	[-0.14, 0.17]	0.06	0.07	0.77	[-0.08, 0.19]
Father's education	-0.03	0.13	-0.23	[-0.29, 0.23]	-0.19	0.12	-1.64	[-0.42, 0.04]
Father's age (years)	0.01	0.04	0.20	[-0.07, 0.08]	0.06	0.03	1.72	[-0.01, 0.13]
Father's psychological capital (X)	0.12	0.06	1.97^{*}	[0.001, 0.23]	0.49	0.03	14.12^{***}	[0.42, 0.55]
Housing type (W)	1.10	0.08	13.48^{***}	[0.94, 1.27]	-	-	-	-
X × W (interaction)	0.18	0.08	2.16^{*}	[0.02, 0.34]	-	-	-	-
Community social capital (M)					0.38	0.04	11.06^{***}	[0.32, 0.45]
R^2	0.51				0.60			
F	63.87^{***}				105.14^{***}			

Table S4. Moderated mediation analysis: Housing type as the moderator for Mother's psychological capital.

Variables	Model 1: Community social capital (M)				Model 2: Adolescent psychological capital (Y)			
	β	*SE*	*t*	95% CI	*β*	*SE*	*t*	95% CI
Gender	0.11	0.06	1.72	[-0.02, 0.23]	-0.02	0.06	-0.42	[-0.14, 0.09]
Grade	0.11	0.09	1.18	[-0.07, 0.29]	-0.10	0.08	-1.21	[-0.26, 0.06]
Adolescent age (years)	0.02	0.08	0.22	[-0.14, 0.17]	0.06	0.07	0.94	[-0.07, 0.20]
Mother's education	0.02	0.08	0.28	[-0.14, 0.19]	-0.08	0.07	-1.13	[-0.23, 0.06]
Mother's age (years)	-0.01	0.02	-0.52	[-0.05, 0.03]	0.01	0.02	0.52	[-0.03, 0.05]
Mother' psychological capital (X)	0.14	0.06	2.45*	[0.03, 0.26]	0.50	0.03	14.70***	[0.43, 0.57]
Housing type (W)	1.11	0.08	13.66***	[0.95, 1.27]	-	-	-	-
X × W (interaction)	0.13	0.08	1.55	[-0.03, 0.29]	-	-	-	-
Community social capital (M)					0.38	0.03	11.16***	[0.31, 0.45]
R^2	0.51				0.61			
F	63.30***				109.37***			

Table S5. Moderated mediation analysis: Ring-road residential location as the moderator for Father's psychological capital.

Variables	Model 1: Community social capital (M)				Model 2: Adolescent psychological capital (Y)			
	β	*SE*	*t*	95% CI	*β*	*SE*	*t*	95% CI
Gender	0.12	0.07	1.66	[-0.02, 0.27]	0.01	0.06	0.13	[-0.11, 0.12]
Grade	0.002	0.11	0.02	[-0.22, 0.22]	-0.13	0.09	-1.49	[-0.30, 0.04]
Adolescent age (years)	0.04	0.09	0.43	[-0.14, 0.22]	0.06	0.07	0.77	[-0.08, 0.19]
Father's education	-0.11	0.15	-0.73	[-0.41, 0.19]	-0.19	0.12	-1.64	[-0.42, 0.04]
Father's age (years)	0.04	0.04	0.80	[-0.05, 0.12]	0.06	0.03	1.72	[-0.01, 0.13]
Father**'s** psychological capital (X)	0.59	0.04	14.88***	[0.51, 0.66]	0.49	0.03	14.12***	[0.42, 0.55]
Ring-road residential location (W)	-0.07	0.04	-1.70	[-0.14, 0.01]	-	-	-	-
X × W (interaction)	0.05	0.04	1.40	[-0.02, 0.13]	-	-	-	-
Community social capital (M)					0.38	0.04	11.06***	[0.32, 0.45]
R^2			0.33				0.60	
F			30.36***				105.14***	

Table S6. Moderated mediation analysis: Ring-road residential location as the moderator for Mother's psychological capital.

Variables	Model 1: Community social capital (M)				Model 2: Adolescent psychological capital (Y)			
	β	*SE*	*t*	95% CI	*β*	*SE*	*t*	95% CI
Gender	0.09	0.07	1.19	[-0.06, 0.24]	-0.02	0.06	-0.42	[-0.14, 0.09]
Grade	0.03	0.11	0.26	[-0.18, 0.24]	-0.10	0.08	-1.21	[-0.26, 0.06]
Adolescent age (years)	0.05	0.09	0.56	[-0.13, 0.23]	0.06	0.07	0.94	[-0.07, 0.20]
Mother's education	0.02	0.09	0.21	[-0.17, 0.21]	-0.08	0.07	-1.13	[-0.23, 0.06]
Mother's age (years)	-0.01	0.03	-0.43	[-0.06, 0.04]	0.01	0.02	0.52	[-0.03, 0.05]
Mother' psychological capital (X)	0.59	0.04	14.72**	[0.51, 0.66]	0.50	0.03	14.70***	[0.43, 0.57]
Ring-road residential location (W)	-0.07	0.04	-1.67	[-0.14, 0.01]	-	-	-	-
X × W (interaction)	0.06	0.04	1.55	[-0.02, 0.13]	-	-	-	-
Community social capital (M)					0.38	0.03	11.16***	[0.31, 0.45]
R^2	0.32				0.61			
F	29.56***				109.37***			
